# Computed tomography and magnetic resonance imaging findings in gouty arthritis involving large joints of the upper extremities

**DOI:** 10.1186/s12880-022-00894-3

**Published:** 2022-09-14

**Authors:** Yuling Yang, Yongfei Guo, Shuiquan Yu, Bin Zou

**Affiliations:** 1 Guangdong, Department of Radiology, Zhongshan Hospital of Traditional Chinese Medicine Affiliated with Guangzhou University of Chinese Medicine, Zhongshan, 528400 Guangdong People’s Republic of China; 2 Guangdong, Department of Ultrasonography, Zhongshan Hospital of Traditional Chinese Medicine Affiliated with Guangzhou University of Chinese Medicine, Zhongshan, 528400 Guangdong People’s Republic of China

**Keywords:** Gouty arthritis, Computed tomography, Magnetic resonance imaging, Upper extremities

## Abstract

**Background:**

We aimed to analyze the computed tomography (CT) and magnetic resonance imaging (MRI) findings of gouty arthritis primarily involving the large joints of the upper limbs, signal or density characteristics of the tophi, growth patterns, involvement of the adjacent joints, and differentiation from other lesions occurring in this area and to discuss the causes of misdiagnosis.

**Methods:**

CT and MRI data were collected from 14 patients with gouty arthritis, primarily involving the shoulder and elbow joints, and their imaging features were analyzed.

**Results:**

All the patiens were ranged from 28-85 years old, and the tophi deposition can be observed on either CT or MRI.The tophi deposition apperas as slightly higher density nodules or masses on CT images,or nodules or masses on MRI with isosignal/hypointensity on T1WI and hyperintensity on T2WI. Five patients showed narrowing of the affected joint space, four had different degrees of bone erosion under the articular surface, eight developed joint effusion, and all showed surrounding soft tissue swelling. The tophi grew around the joint, with anterolateral and posterolateral tophi predominantly in the shoulder joint and dorsal tophi predominantly in the elbow joint on the MRI, with compression and edema of the surrounding soft tissues.

**Conclusions:**

Gouty arthritis occurs in the large joints of the upper limbs and is characterized by fluid accumulation in the joint capsule and the formation of tophi. These tophi are usually large, with subcutaneous bone resorption and erosion, with or without cartilage destruction. However, extensive edema appeared in the soft tissue around the tophi, but the edema only produced pressure without any obvious signs of soft tissue infiltration, which may be distinguished from the joint tumor. In addition, the gout incidence rate is increased in young patients. Therefore, when the patient has a large joint mass, it is important to confirm whether there is a history of gout.

## Background

Gout is a rheumatic metabolic disease. Hyperuricemia is accompanied by urate deposition, leading to gouty arthritis, uric acid nephropathy, and kidney stones. The gout incidence rate is gradually increasing in China [1], low in some parts of the world, and relatively high in coastal or economically developed areas, which is related to dietary structure changes [2–4]. It is clinically divided into acute, intermittent, and chronic gouty stone phases. Patients with gouty arthritis usually experience joint pain and limited movement in the corresponding area due to joint inflammation and bone marrow edema. Gouty arthritis in the spine may be associated with hypertension, obesity, impaired renal function, or poorly controlled hyperuricemia [5–8]. It is a type of metabolic disease in which sodium urate crystals are deposited in the bone and joints due to the supersaturation of uric acid in the blood, causing local inflammatory reactions and bone tissue destruction. Gouty stone formation is the pathological basis of gouty arthritis, usually characterized by nodular tophi, mostly involving the ankle, knee, and other extremity joints, and most often involving the intercuneiform joints, with bone resorption and erosion under the joint surface and narrowing of the joint space. Gouty arthritis involving large joints of the upper extremities is rare in clinical practice. It is very rare for gout to affect the shoulder and elbow joints. In the previously retrieved case reports, gout stones were mainly deposited around the joints, which rarely caused bone erosion under the joint surface [9–11]. It is often misdiagnosed in clinical work, thus delaying treatment or not being finally confirmed.

To correctly diagnose gouty arthritis occurring in the large joints of the upper limbs, we collected 14 cases of gouty arthritis involving the elbow and shoulder joints of the upper extremities that were clinically or pathologically diagnosed in our hospital, compiled their clinical and imaging data, analyzed their imaging manifestations, and discussed the causes of misdiagnosis and how to differentiate them from other lesions. Gouty arthritis most commonly affects the joints of the extremities, including the joints of the first toe, metatarsal, intercuneiform, ankle, knee, wrist, and elbow. It often affects the joints unilaterally and predominantly occurs as osteoarthritic lesions, with uric acid deposition in the joints or bursae as the pathological basis, leading to surrounding soft tissue inflammation and a slow erosion of the bone surface, resulting in bone resorption and destruction from the outside to the inside.

## Methods

### Patients

General data: A total of 14 patients with gouty arthritis involving the large joints of the upper extremities were admitted to the Zhongshan Chinese Medicine Hospital from April 2014 to April 2021. The requirement for informed consent from the patients was waived due to the study's retrospective nature. All patients were males, with an age range of 28–85 years. Seven of the 14 patients underwent computed tomography (CT), and seven underwent magnetic resonance imaging (MRI). Among the eight patients with gout in their elbow joint, six underwent MRI and two underwent CT; among the six patients with gout in their shoulder joint, only one underwent an MRI and five underwent CT.

### Data collection

CT or MRI and clinical data, including uric acid levels, pathological findings, and medical history, were collected from all patients to confirm whether they had gout.

### Image acquisition

*MRI examination* Siemens 1.5T MR imager with a small joint array coil was used. The MR scan sequence included sagittal T2-weighted imaging (WI), T1WI fat suppression of the spine, and axial T2WI (T1WI: TR 300 ms and TE 4 ms; T2WI: TR 1200 ms and TE 84 ms; axial FOV, 139 mm × 139 mm; matrix, 512 × 512; layer thickness, 4 mm; layer spacing, 5 mm).

*CT examination* GE 16-row spiral CT or Siemens 64-row 128-layer spiral CT was used for the imaging with a layer thickness and spacing of 2 mm. After completing the scan, the original images were post-processed. Multiplanar reformation coronal and sagittal reconstructions were performed, and the reconstructed layer thickness and spacing were 2 mm.

### Medical image feature analysis

Two radiologists with 10 years of work experience reviewed and evaluated the image data. Specific imaging evaluation indicators included the site of gouty arthritis, gouty stone growth size, morphology, density, signal characteristics, subsurface bone changes, whether the joint space was narrowed, surrounding soft tissue conditions, and whether there was fluid accumulation in the joints. The gout stones size were between 2 to 40 mm. The number of patients with multiple gout stones was 14/14 (100%), and the gout stones were mainly strip-shaped in 4/14 (29%), nodular in 5/14 (36%), and lumpy in 5/14 (36%) cases, with uneven density or signal in 14/14 (100%), bone absorption under the joint surface in 4/14 (29%), narrowed joint space in 5/14 (36%), swollen surrounding soft tissue in 14/14 (100%), and joint effusion in 8/14 (57%) cases (Table 1).

**Table 1:** CT or MRI features and pathological basis of 13 patients

Image feature	n	%	Pathology
Tophi	14	100	Urate crystals
Joint fluid	8	57	Effusion
Narrowing of joint space	5	36	Urate crystals form tophi and erosion of the cartilage
Intraarticular bone erosion	4	29	Urate crystals form tophi and erosion of the bone
Swelling of surrounding soft tissue	14	100	Soft tissue edema

### Statistical treatment

Statistical processing data were processed with the SPSS 25.0 statistical software. The counting data were expressed in cases or percentages, and the comparison between groups was conducted using the χ^2^ test; *P* < 0.05 means the difference was statistically significant.

## Results

All patients were confirmed to have gout based on the clinical, pathologic, radiographic, or laboratory examination; all patients underwent either CT or MRI. The results of blood uric acid were from 303 to 688. This group of cases showed no positive correlation between uric acid and the tophi size (Table 2).

**Table 2:** Scan parameters and basic information of patients

Sex	Age	Blood uric acid level	Location	Diagnostic method	Image examination method
M	63	503	Left shoulder joint	Surgical pathology	MRI
M	28	587	Left elbow	Surgical pathology	MRI
M	55	591	Right elbow	Surgical pathology	MRI
M	51	519	Left elbow	Surgical pathology	CT
M	44	666	Right shoulder joint	Surgical pathology	CT
M	66	711	Right elbow	Surgical pathology	MRI
M	51	562	Left elbow	Surgical pathology	MRI
M	47	621	Left elbow	Surgical pathology	MRI
M	56	587	Right elbow	Surgical pathology	MRI
M	45	630	Double shoulder joint	Years of gout history + gouty arthritis in other parts	CT
M	63	688	Double shoulder joint	Years of gout history + gouty arthritis in other parts	CT
M	85	303	Double shoulder joint	Years of gout history + gouty arthritis in other parts	CT
M	49	567	Double shoulder joint	Years of gout history + gouty arthritis in other parts	CT

**Fig. 1:**
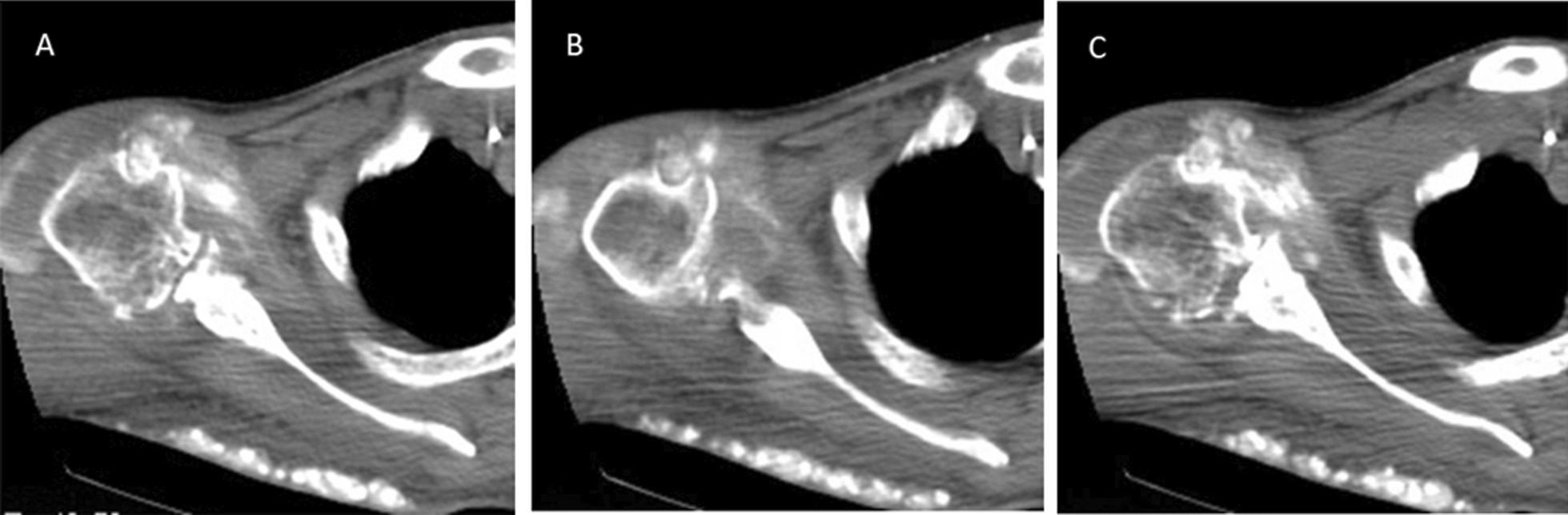
A 50-year-old male patient who underwent shoulder joint CT and showed multiple high-density gout nodules around the joint, with joint space stenosis and bone absorption under the joint surface. Multiple high-density gout nodules can also be seen under the back

CT or MRI directly demonstrated tophi deposition. The seven patients with tophi who underwent CT showed slightly high-density strip-shaped or nodular of different sizes, without (3/7) or with (4/7) different degrees of narrowing of the adjacent joint space, three patients were accompanied by varying degrees of bone erosion under the joint surface, with some of the larger tophi being mass-like, compressing the adjacent soft tissues (Fig. 1A–C), and one patient showed joint effusion. The seven patients who underwent MRI showed nodular or mass-like abnormal signal shadows, with isosignal/hypointensity on T1WI and hyperintensity on T2WI, varying in size and morphology, and some of the larger tophi had mixed signals on T1WI and T2WI. One patient showed narrowing of the affected joint space and a little bone erosion on the joint surface. Tophi grew around the joint, and they were predominantly observed on the anterior and lateral sides in the shoulder joint and on the dorsal side in the elbow joint. Compression and edema of the surrounding soft tissues were observed on the MRI. All seven patients showed joint effusion.

There was no significant difference between CT and MRI patients in showing gout stone, narrowing of joint space, intraarticular bone erosion, and surrounding soft tissue swelling (*P* > 0.05). However, for the display of joint effusion, six of the seven patients who underwent MRI examination were positive, and only Only two of seven patients who underwent CT examination were positive There was a statistical difference between the two groups of patients (*P* < 0.05).

## Discussion

### Imaging features

The imaging diagnostic methods of gouty arthritis include X-ray scan, CT, and MRI. Radionuclide imaging and X-ray scan are the preferred imaging examination methods for clinical diagnosis and screening of gouty arthritis, but the diagnostic value for early gouty arthritis and differential diagnosis with other types of arthritis is limited. CT is better than X-ray scan in detecting urate crystals, gout stones, and joint bone resorption. MRI has obvious advantages in detecting joint synovium and surrounding soft tissue lesions. Therefore, both CT and MRI can be used to detect early gouty arthritis. However, because gouty arthritis involving large joints of upper limbs is very rare, and only a few related cases have been reported clinically [12–14], there are few manuscripts on the imaging analysis of the elbow and shoulder gouty arthritis. Therefore, we collected the clinical, laboratory, and radiological information on gouty arthritis involving elbow and shoulder joints and summarized the CT and MRI of gouty arthritis originating from the large joints of the upper limbs. The patients in the study showed bone resorption and erosion under the joint surface, accompanied by high-density or iso-T1, slightly longer T2, and gouty stone formation around the joints. The data showed no positive correlation between the tophi size and the value of blood uric acid.

Some cases of our study occurred in the elbow joint and were misdiagnosed as tumor lesions due to the formation of soft tissue masses and erosion of the adjacent bones. Patients with gouty arthritis occurring in the shoulder joint had tophi deposited in and around the joint space, involving the tendons around the shoulder joint or causing joint space narrowing, resulting in movement restriction of the shoulder joint and other symptoms extremely similar to those of frozen shoulder. These symptoms are similar to those of a frozen shoulder, such as limitation of the shoulder movement, leading to incorrect diagnosis at the initial consultation. Moreover, regardless of whether the shoulder or elbow joint was involved, inflammation of the joint surface caused the patient to experience pain in the corresponding area.

### Analysis of the causes of misdiagnosis

**Fig. 2:**
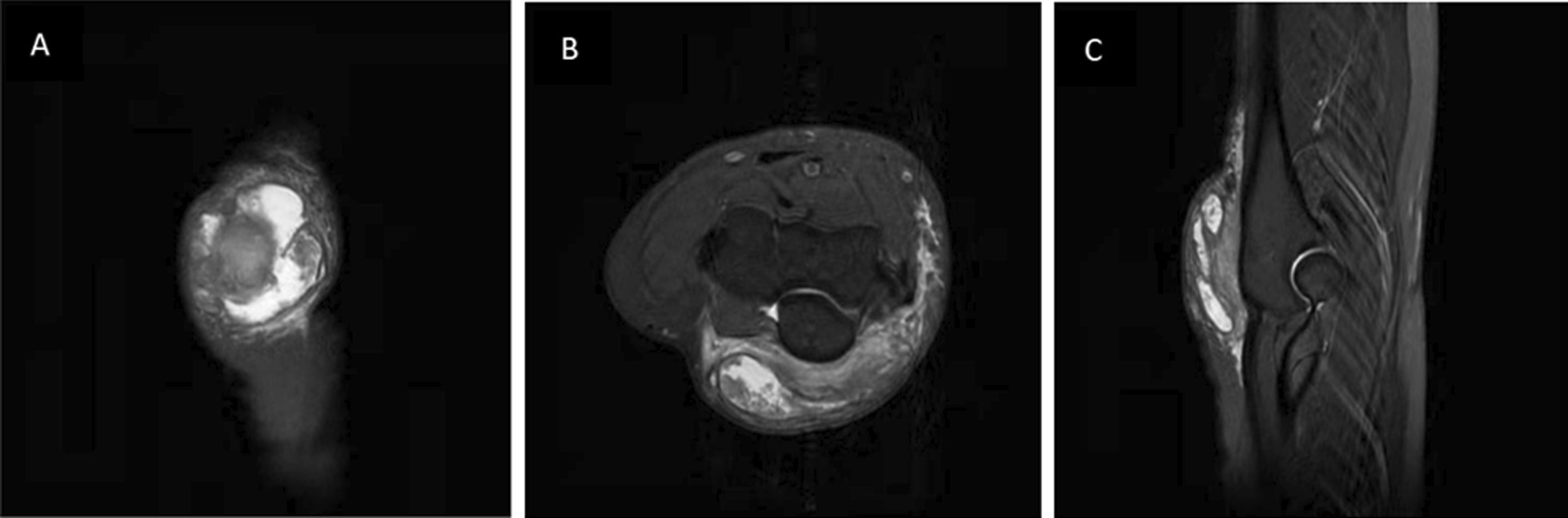
A 28-year-old male patient who underwent an MRI of the elbow joint and showed a huge mass with mixed signals, extensive edema of the surrounding soft tissues, and bony involvement of the adjacent joints. **A** T2-FS coronal, **B** T2-FS transverse, **C** T2-FS sagittal

**Fig. 3:**
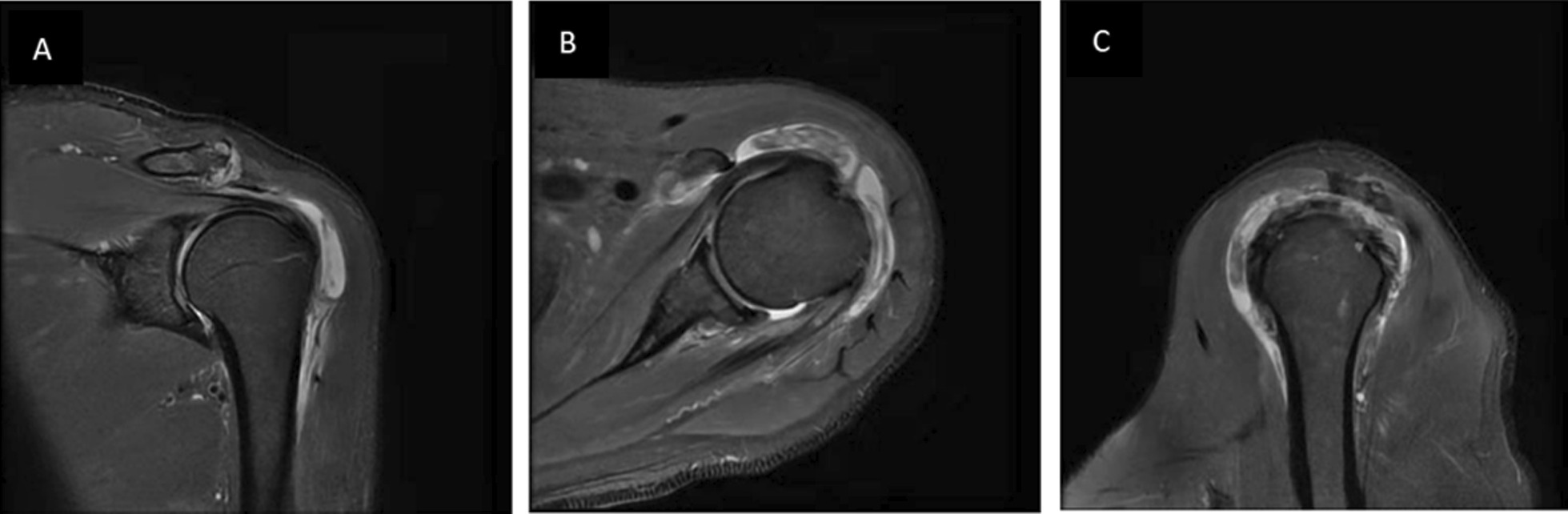
A 63-year-old male patient who underwent shoulder joint MRI and showed no obvious signs of soft tissue invasion around tophi. **A** T2-FS coronal, **B** T2-FS transverse, **C** T2-FS sagittal

The gold standard for diagnosing gout is urate crystals in the patient's joint fluid or gout stone under the microscope. When the patient's blood uric acid is more than 420 µmol/L and there is characteristic arthritis, urinary tract stone, or renal colic, the diagnosis of gout can also be considered clinically [15]. Among the 14 patients in this study, only six were accurately diagnosed after the first imaging examination, namely, five patients who underwent a CT scan of the shoulder joint and one who underwent an MRI of the elbow joint with clear tophi; the remaining eight were misdiagnosed. The reasons for misdiagnoses were analyzed, among which three patients who underwent an MRI of the elbow joint were considered to have a large mass at the first examination site, where the MRI demonstrated a huge mass with mixed signals and extensive edema of the surrounding soft tissues. Two had no abnormal adjacent joint bone (Fig. 2A–C). However, the postoperative retrospective analysis revealed that the soft tissues around the tophi showed extensive edema, but the edema applied pressure only without any obvious signs of soft tissue invasion (Fig. 3A–C). The remaining five patients were diagnosed with synovial inflammation of the joints only. Misdiagnosis occurred due to the tophi's small size, and the patients reported no clear history of gout; therefore, the possibility of this disease was not fully considered at the initial diagnosis. However, the postoperative retrospective analysis demonstrated powder lines involving joints around the tophi with slight bone resorption and narrowing of the joint space, which, combined with specific laboratory tests, could be consistent with gouty arthritic changes.

### Differential diagnosis

The following differential diagnoses can be considered for patients demonstrating the signs and symptoms of gouty arthritis:

1. *Traumatic arthritis* Patients with traumatic arthritis usually have a clear history of trauma. The lesion can be observed months or years after the trauma, which presents with narrowing of the injured joint space, accompanied by the formation of a bony flap at the joint edge, with a higher density in the flap than the tophi, and a more lamellar rather than mass-like morphology, which may be surrounded by free body formation, with or without joint space and cartilage damage.
2. *Ossifying myositis* The early stage of ossifying myositis is the stage of trauma and inflammatory reaction, often manifesting as significant muscle, fascia, and tendon edema without an exact mass. The middle stage is the most difficult to diagnose, and the typical imaging manifestation is the reduction of lesion edema on CT and MRI, with progressively clearer borders and stratification signs. Finally, in the late stage, with the advancement of calcification and ossification, the lesion tends to be completely ossified, the edema around the lesion disappears, the boundary is clear, and the lesion appears as a strip or irregularly shaped calcified foci and bone masses, and the CT shows irregularly shaped or strip-shaped high-density bone masses, and the MRI shows iso-T1WI and iso-T2WI signal changes, and the signal is the same as that of the adjacent bone; there are no signs of erosion of the surrounding muscles and soft tissues; the lesion is often not connected to the adjacent bone, and there is no destruction of the adjacent bone or periosteal reaction.
3. *Frozen shoulder *Frozen shoulder usually occurs in middle-aged and elderly patients with shoulder joint degeneration. Frozen shoulder is caused by vasoproliferative synovitis of the joint capsule, followed by fluid leakage. Inflammatory factors and proteins from the joint cavity cause fibrosis, scar formation, and thickening of the joint capsule and other soft tissue structures. Images show thickening and edema of the joint capsule, followed by a smaller joint cavity, which is distinct from gouty arthritis, characterized by fluid accumulation in the joint capsule and urate crystal deposits in the joint cavity or the periarticular tissues, causing an inflammatory response. We can make a diagnosis if the image shows the tophi.

### Treatment methods

The range of drug treatment options for acute and chronic gout has changed dramatically over the last 20 years [16]. Medication such as colchicine is the primary treatment for patients with gouty arthritis involving large joints in the upper extremities to reduce uric acid concentrations combined with the dietary intervention [17]. However, some patients may be admitted to the hospital with no obvious symptoms and only with swelling around the joint, which requires imaging combined with laboratory tests to arrive at an accurate diagnosis and provide targeted interventions to prevent possible future symptoms [18–23]. In addition, surgery such as arthrotomy and aspiration, lesion removal, and joint replacement may be planned according to the patient's condition to prevent more serious bone destruction of the joints caused by tophi deposition [17].

### Limitations

**Fig. 4:**
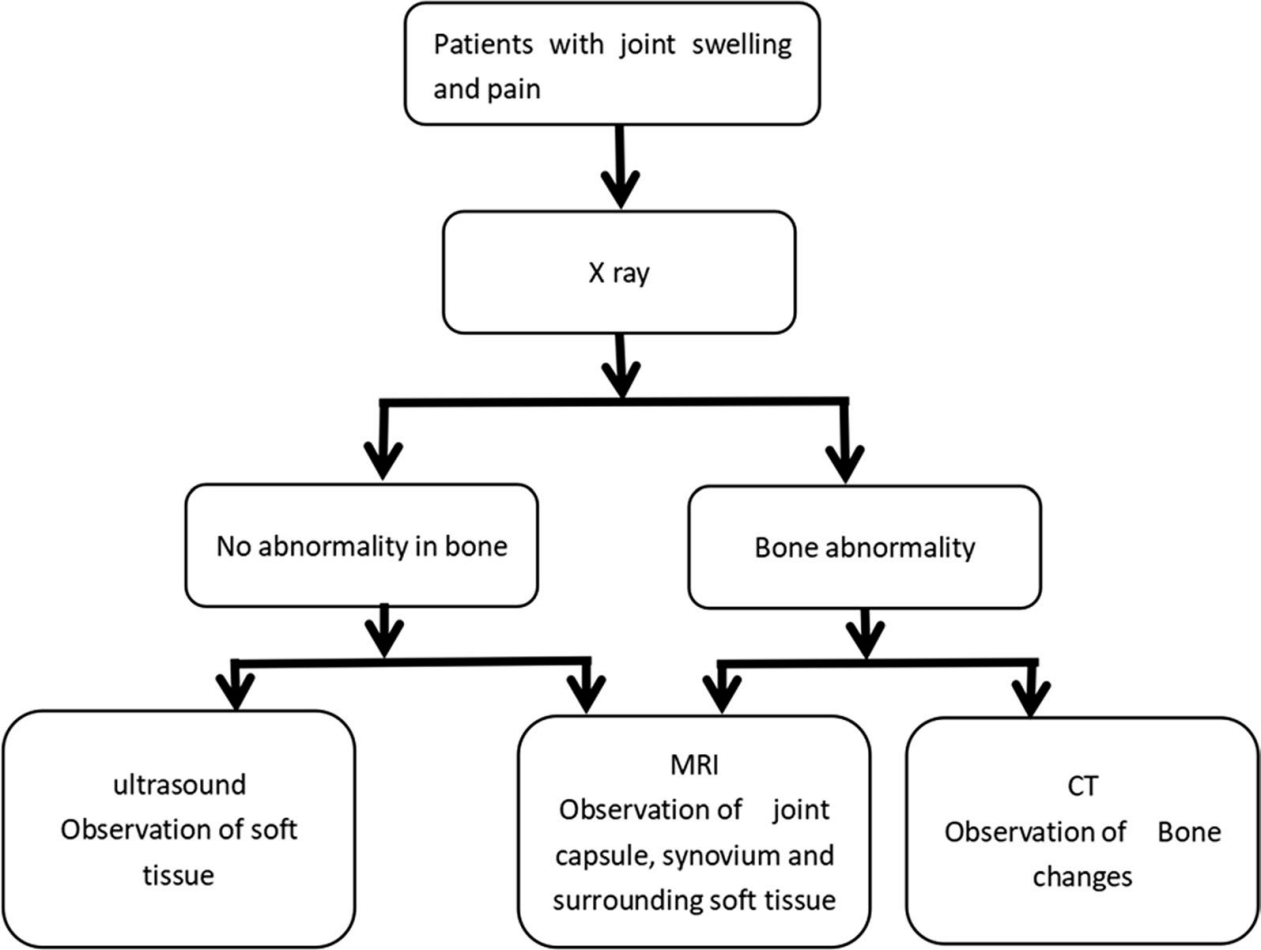
Diagnostic flow-chart

CT is not sensitive to showing synovial hyperplasia and minute tophi formation in the early stage of gouty arthritis; MRI is sensitive to showing soft tissue and bone abnormalities of gout, although imaging findings are not specific [24]. Therefore, we believe that MRI in the early stage of gouty arthritis has better sensitivity, and CT in the middle and late stage of observation of joint bone changes is better (Fig. 4). When the uric acid and pain of the patient's upper limb joint increases, we can combine the above two imaging examinations and observations to make a correct judgment on the early gouty arthritis of the upper limb joint.

## Conclusions

The imaging manifestations of gouty arthritis involving the large joints of the upper limbs have certain characteristics. In this group of cases, when the elbow and shoulder joints are involved, there is fluid accumulation in the surrounding joint capsule, and gout stones are formed. These stones are usually large at the time of diagnosis, accompanied by subsurface bone resorption and erosion, with or without cartilage destruction. When we fully understand the above characteristics and combine the differential diagnosis of gouty arthritis, we can strive to improve the accuracy of imaging diagnosis, provide clinical recommendations and early treatment, and avoid the serious consequences caused by delayed treatment.

## Data Availability

The datasets used and/or analyzed during the current study are available from the corresponding author on reasonable request.

## Abbreviations

CT: Computed tomography
MRI: Magnetic resonance imaging
WI: Weighted imaging
